# Identifying, assessing and responding to perpetration of domestic abuse: practice guide for mental health professionals

**DOI:** 10.1192/bja.2024.39

**Published:** 2025-01

**Authors:** Philippa Greenfield, Marilia Calcia, Chris McCree, Maneek Sahota, Holly Thomas, Kyla Kirkpatrick, Rebecca Vagi, Louise M. Howard, Sarah Markham, Vishal Bhavsar

**Affiliations:** Consultant psychiatrist and named doctor for adult safeguarding at Camden and Islington NHS Foundation Trust, London, UK, and the Royal College of Psychiatrists’ co-presidential lead for women and mental health, London, UK.; Consultant psychiatrist at South London and Maudsley NHS Foundation Trust, London, UK, and researcher with the Section of Women's Mental Health at the Institute of Psychiatry, Psychology & Neuroscience, King's College London, UK.; Lead for parental mental health with the Helping Families Team and Perinatal Community Services at South London and Maudsley NHS Foundation Trust, London, UK.; Safeguarding and domestic abuse practitioner at Camden and Islington NHS Foundation Trust, London, UK.; Domestic abuse prevention coordinator at Central and North West London NHS Foundation Trust, London, UK.; Director of the Drive Partnership, London, UK.; National lead for Make a Change at Respect, London, UK.; Emeritus Professor of Women's Mental Health at the Institute of Psychiatry, Psychology & Neuroscience, King's College London, UK.; Visiting researcher at the Institute of Psychiatry, Psychology & Neuroscience, King's College London, UK, and Patient Reviewer for the Quality Network for Forensic Mental Health Services at the Royal College of Psychiatrists, London, UK.; Research fellow with the Section of Women's Mental Health at the Institute of Psychiatry, Psychology & Neuroscience, King's College London, UK, and an honorary consultant in forensic and general adult psychiatry at South London and Maudsley NHS Foundation Trust, London, UK.

**Keywords:** Domestic abuse, violence, risk assessment

## Abstract

Domestic abuse – abusive behaviour perpetrated by an adult towards another adult to whom they are personally connected (e.g. partners, ex-partners or family members) – damages mental health, increases mental health service use and challenges clinical management. Training and guidance for mental health professionals on identifying and responding to patients exposed to domestic abuse are available, but there has been less development of resources for mental health professionals in identifying, assessing and responding to perpetrators of domestic abuse. In this article, we describe a framework for responding to domestic abuse perpetration in clinical settings in general adult mental health services, aimed at improving practice. This could support mental health professionals in sensitive enquiry and assessment for domestic abuse perpetration, and guide appropriate responses, as part of routine training and continuing professional development.

## LEARNING OBJECTIVES

After reading this article you will be able to:

- understand current evidence on the association of domestic abuse perpetration with mental ill health and mental health service use
- appreciate the role of mental health professionals in the identification, assessment and response to perpetration of domestic abuse
- consider the role of mental health interventions in responding to perpetration of domestic abuse in mental health services.

Domestic abuse is a prevalent and costly public health and clinical problem. It has a negative impact on mental and physical health and is associated with greater use of primary and secondary healthcare for mental health conditions in people who are subject to domestic abuse as victims/survivors. UK law passed in 2021 defines domestic abuse as abusive behaviour perpetrated by a person towards another person who is personally connected to the perpetrator, where they are both over 16 years of age. Abusive behaviour includes physical, sexual, psychological and economic abuse, and threatening, coercive and controlling behaviour. The law considers two people ‘personally connected’ where they are or have been engaged, married or in civil partnership, in an intimate relationship, in a parental relationship to the same child, or are related. Statutory guidance highlights the role of health services in identifying domestic abuse and recognises that children who are related to the (adult) perpetrator or victim/survivor of domestic abuse, and who see, hear or otherwise experience the impact of domestic abuse, are also victims/survivors (Home Office 2022). Harmful behaviour perpetrated by adult children towards parents is also included in the legal definition of domestic abuse.

## Perpetration of domestic abuse and mental health services

Lifetime domestic abuse victimisation is reported by 1 in 3 women and 1 in 4 men globally (UN Women 2020), although repeated and severe domestic abuse victimisation is more commonly experienced by women. Domestic abuse is associated with mental health problems, including depression, post-traumatic stress disorder, anxiety disorders and psychoses (Oram 2022). According to Office for National Statistics data for England and Wales for the year ending March 2022, 46% (*n* = 84) of adult female homicide victims were killed in a domestic homicide, compared with 11% (*n* = 50) of adult male homicide victims (Office for National Statistics 2022).

People who are in contact with mental health services are at higher risk of both experiencing and perpetrating domestic abuse compared with the general population. A study of Swedish psychiatric registers linked to criminal justice data on arrests for intimate partner violence perpetrated by men against women found increased rates of intimate partner violence perpetration among men diagnosed with all psychiatric disorders except autism (Yu 2019). In sibling comparisons that took account of genetic and environmental characteristics shared within families, individuals with diagnoses of depressive disorder, anxiety disorder, alcohol use disorder, drug use disorder, attention-deficit hyperactivity disorder and personality disorders had a 1.7- to 4.4-fold higher risk of intimate partner violence perpetration than their unaffected siblings.

Mental ill health and mental health service contact are common in the personal histories of people who perpetrate domestic homicide in England and Wales. One analysis of 141 publicly available domestic homicide reviews found that 49% of perpetrators had a mental health diagnosis (Chantler 2020). Another reported that perpetrators of domestic homicide in England and Wales who had made contact with mental health services leading up to the offence were significantly more likely than those without mental health service contact to have a history of substance misuse (69% *v.* 50%), criminal justice system contact (78.9% *v.* 60%) and attempted suicide (62.2% *v.* 15%) (MacInnes 2023). A review of London domestic homicides reported that mental health problems were identified in 64% of perpetrators of adult family homicides and 44% of perpetrators of intimate partner homicides (Montique 2019). At a general population level in England, in a recent analysis of household survey data from 2014, the community prevalence of self-reported perpetration of intimate partner violence was around 8%, and people who had reported intimate partner violence perpetration were nearly three times more likely to have used mental health services in the previous year compared with those who had not (Bhavsar 2023).

Responding to perpetration of domestic abuse is therefore highly relevant to mental health services, although this has not been of previous focus in service/guideline development, policy-making and research. Clinical responses to perpetration of domestic abuse may involve balancing therapeutic interventions with duties to protect the patient and public, which may make professionals uncertain about how to respond. In this article we therefore draw on existing practice and research evidence to provide up-to-date guidance on how mental health professionals should respond to the perpetration of domestic abuse, with a focus on identification, assessment and response. We acknowledge that the topic of domestic abuse is sensitive, and terminology on this topic risks stigmatising patients. People perpetrating domestic abuse will also be at varying levels of recognising and owning their behaviours as domestic abuse, and of motivation for help-seeking.

To align with existing legislation in England and Wales, policy and guidance we use the following terms in this paper: perpetrator/perpetration of domestic abuse and domestic abuse perpetrator/perpetration. We use ‘victim/survivor’ to refer to those subjected to ongoing or past domestic abuse.

## The aim and development of this guide

We aim to provide a framework for professionals to improve professional practice in identifying and responding to perpetration of domestic abuse by people who have contact with mental health services.

To develop this guide, we (a group of mental health clinicians, domestic abuse practitioners and academics) met to review the following sources: information/guidance available on the web on assessing and managing violence in the UK's National Health Service (NHS) mental health services; mental health professional guidance on identifying, assessing and managing domestic abuse, some of which refers also to perpetrators (Yapp 2018); and guidance on the identification, assessment and management of perpetrators of domestic abuse from organisations supporting specialist interventions, including those providing behaviour change programmes to support perpetrators to reduce/stop their abusive behaviour to improve victim/survivor safety (Respect 2022; Home Office 2023).

We hope this article can be integrated into local mental health trust/health board policies on safeguarding, domestic abuse and violence risk management. Primarily we focus on clinical practice with working-age adults (16–64 years) with mental health conditions seen in secondary mental health services – clinical responses to children (under the age of 16) perpetrating domestic abuse towards (adult) parents are therefore not covered in this article; however, we do consider violence from adults towards their parents. Mental health professionals should receive training in responding to perpetration of domestic abuse. The material presented in this guide should be considered alongside local safeguarding and domestic abuse training, policy and procedures.

## Overview and rationale

**FIG 1:**
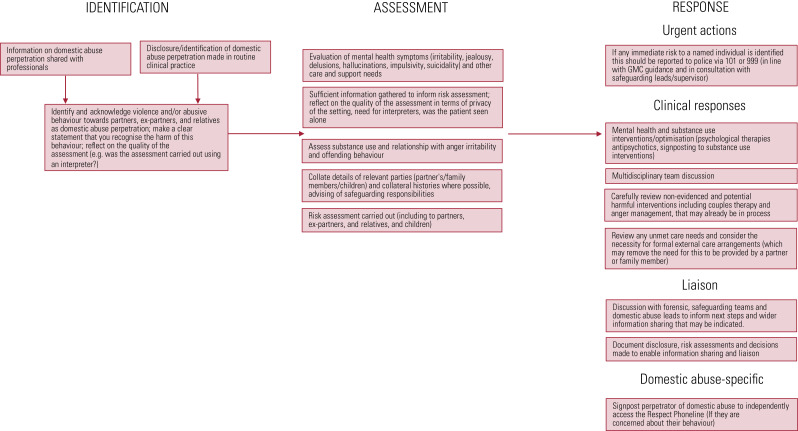
Framework for identifying, assessing and responding to perpetration of domestic abuse. GMC, General Medical Council.^a^ a. General Medical Council (GMC) (2015).

Clinical responses to domestic abuse perpetration are important because domestic abuse is highly prevalent and mental health professionals can have an important opportunity to identify and respond appropriately to patients who may be causing harm to others (Table 1). In this article we present a framework (Fig. 1) to guide clinical responses to domestic abuse perpetration in mental health services. We describe:

- typical contexts for the disclosure/identification of domestic abuse perpetration in clinical practice
- guidance on the clinical approach to talking with a patient who may be perpetrating domestic abuse
- clinical indicators in the psychiatric history that might suggest specific enquiry about domestic abuse perpetration
- assessment strategies
- clinical and risk management in domestic abuse perpetration in mental health services.

**TABLE 1:** Rationale: why improve practice in identifying and responding to domestic abuse perpetration in mental health services?^a^

Beneficiary	Details
Benefits to the patient	Early identification of risk factors for further violence Improved access to domestic abuse perpetrator interventions, including help-seeking services such as the Respect Phoneline, behaviour change work and case management where this is available Identification of socioeconomic risks, e.g. to tenancy or employment, and intervention to reduce these risks Improved family functioning in some cases, e.g. maintained contact with children Potential to strengthen therapeutic relationship, reduce distress/shame through enabling honest dialogue and access to support
Benefits to family/carers/partners/survivors	Improved mental health and well-being of victims/survivors of domestic abuse Reassurance to victims/survivors and other family members that risks posed by the perpetrator are being addressed Where a perpetrator is supported to access a behaviour change or case management programme that meets the Respect and/or Home Office accreditation standards for perpetrator interventions, this will include the offer of integrated support for victims/survivors. This assists victims/survivors in accessing services for themselves, which can increase their ability to make more informed choices and increase their safety
Benefits to other members of the public	Domestic abuse perpetration is correlated with other forms of violent behaviour (e.g. community violence), and prompt identification and management can reduce later risks to the public. Health professionals are part of a coordinated community response to domestic abuse to secure the safety of victims/survivors and hold abusers to accoun.
Benefits to their children	Interventions and support to children and the family aim to reduce further harm. They may also have the potential to reduce longer-term impact on children's health and development and the higher risk that these children will themselves perpetrate domestic abuse when they reach adulthood

a The potential benefits displayed in this table are based on our discussion of clinical experience and some limited available research evidence (described elsewhere in the article). More evidence is needed on outcomes associated with responses to domestic abuse perpetration in mental health services.

## Clinical enquiry and exploration of domestic abuse perpetrators in mental health services

### Contexts for the disclosure/identification of domestic abuse perpetration

People perpetrating domestic abuse may not necessarily initiate self-disclosure, but may nevertheless display indicators of their abusive behaviour. Health professionals should understand that domestic abuse includes coercive and controlling behaviours used to control victims/survivors (Crown Prosecution Service 2023). Coercive and controlling behaviours are common and can happen alongside other types of abuse, including physical, sexual and economic abuse. Therefore, it is necessary to apply professional curiosity and sensitivity when asking about current and past relationships, including as part of enquiring about and assessing risk of violence to others. Statements made by individuals about having ‘a temper’ or about ‘problems at home’, ‘volatile’ relationships, jealousy or possessiveness should be assessed in depth. Perpetrators of domestic abuse may harbour a strong sense of grievance, for example referring to themselves as victims/survivors of intimate partner or family abuse, or describing instances where they themselves experienced harm. Responding from a neutral, non-judgemental stance by asking open-ended questions to explore the impacts and how they are managing the situation can avoid colluding with the perpetrator of domestic abuse. Professionals should acknowledge patients’ experiences of personal trauma, including those patients who may be perpetrating harm against others (Oram 2022).

### Example scenarios: how perpetrators might present to mental health services

In Table 2 we present an overview of clinical settings and circumstances where perpetrators of domestic abuse may present as patients. Clinicians should recognise that perpetrators of domestic abuse may also present to mental health services when accompanying a victim/survivor to an appointment. In this situation, the perpetrator may make efforts to prevent the victim/survivor being seen by the clinician on their own. Clinicians should also be wary of claims by people that their partners are mentally ill/symptomatic, which are sometimes made to deflect attention from a perpetrator's responsibility for abuse.

**TABLE 2:** How perpetrators of domestic abuse may present to mental health services^a^

Mental health service setting	Clinical presentation	Issues to consider
Psychiatric liaison assessments in emergency departments	Self-injury, suicidal ideation and suicide attempts, e.g. in the context of relationship problems, anger or abusive behaviour	Obtaining accurate and timely collateral history Acting on reports of violent behaviour in acute situations (e.g. carefully consider a criminal justice mental health referral versus a crisis team pathway)
Places of safety under section 136 of the Mental Health Act	Assessment following an incident of violence and aggression in a public place (e.g. towards members of the public) in the context of violence in intimate and family relationships	Gaining accurate information on the index incident Gaining accurate forensic history Gaining thorough history of current family network and intimate relationships and careful enquiry to explore any risks to these individuals
Community mental health teams	Low mood, anxiety, usually in the context of risks such as self-injury, suicidal thoughts Psychotic symptoms	Disengagement (non-attendance, non-adherence) Careful assessment needed of both family and intimate relationships
Substance use treatment services	Care and treatment for primary substance use disorders	Evaluating relation between substance use pattern and offending/harmful behaviour Balancing risk management and harm reduction strategies
In-patient mental health units	Suicidal thoughts (often, but not always, triggered by relationship problems, separation, etc.) Acts of violence or aggression	How contact with family/partner is managed during in-patient stay and periods of leave An in-patient stay can provide a period of separation that could be important to gain collateral information and offer options for interventions/support to victim/survivor
Forensic mental health services	Risk management of offenders with diagnoses of mental illness (e.g. of personality disorder or psychosis) and with a history of perpetrating domestic abuse (as part of an index offence or otherwise)	Effective multi-agency working (including information sharing) Note that domestic abuse perpetrators may have a non-domestic abuse-related index offence
Memory clinic/mental health of older adults services	Harmful behaviour towards partners/family members by elderly patients with dementia and/or mood disorders	Victims/survivors of domestic abuse perpetrated by people with dementia will often hold caring responsibilities towards the patient
Child and adolescent mental health services (perpetrator aged 16–18)	A child receiving treatment for attention-deficit hyperactivity disorder or depression in CAMHS is reported to have assaulted a parent	Where parents experience harmful behaviour from children under the age of 16, this does not fall into legal definitions of domestic abuse, and this needs discussion with child safeguarding Given the very high prevalence of childhood adversity in CAMHS populations, management planning for CAMHS patients who are causing harm to family members should directly address experiences of trauma and their own current safety from others
Psychotherapy services, including the NHS Talking Therapies programme	Disclosure of perpetration of domestic abuse during psychological treatment, e.g. for common mental disorders or anger difficulties	Balancing therapeutic and risk assessment management responsibilities Note that there is limited evidence to support effectiveness of couples and family therapy in domestic abuse; ongoing interventions received by perpetrators of domestic abuse need to be carefully reviewed and stopped if there is evidence of harm

CAMHS, child and adolescent mental health services; NHS, National Health Service.

a In all assessments ask about children or contact with children. Document names and dates of birth of children. Follow child safeguarding protocols if any risk identified.

### Why should mental health professionals enquire about perpetration of domestic abuse?

Mental health professionals have professional obligations to protect the public, including family members of patients. Effective risk assessment and management is a critical part of good clinical care. Making appropriate enquiries about a patient's use of harmful behaviour is an important part of assessing the context for a patient's presenting complaint (e.g. paranoid thoughts that their partner is having an affair, relationship distress and suicidal ideation), including where there are safeguarding concerns that the patient themselves is vulnerable. Children are considered victims/survivors of domestic abuse where they see, hear or are otherwise affected by domestic abuse among adults (Home Office 2022). Therefore, patients presenting with parenting-related distress should also be asked about relationship safety (both experiences and perpetration of domestic abuse, and the nature of relationships with children), and full information should be collected on children within the family, in consultation with safeguarding teams and domestic abuse teams in the trust/health board, and in line with taking a ‘whole family’ approach in clinical and risk interventions in mental health services (Woodman 2020).

### Indicators that might highlight domestic abuse perpetration

Mental health clinicians should always ask specific questions about domestic abuse (Howard 2017) and should be ready to explore the history more carefully, particularly if any of the following indicators are present. Some of these reflect harmful behaviour and others do not; and some are closely related to, or overlap with, indicators of elevated risk of harm in perpetrators of domestic abuse shown in Box 1.

BOX 1 Factors that indicate greater risk of harm by perpetrators of domestic abuse

- Where the victim/survivor expresses fear of further harm (Heckert 2004)
- Unconscious and other biases on the part of professionals, which may lead to the underestimation, minimisation or collusion in/justification of risks, e.g. cases where families are from a different cultural background from the professional might lead the professional to make assumptions that harmful behaviour is ‘culturally appropriate’ (Richards 2009)
- Where the victim/survivor has attempted to end the relationship (Block 2000)
- Court proceedings relating to child contact or to previous domestic abuse (such as a non-molestation order) (Richards 2009)
- Pregnancy, which is a high-risk period for domestic abuse for the pregnant woman and for the unborn child and any other children in the family (Shah 2010)
- An escalating pattern of domestic abuse (Robinson 2012)
- Stalking behaviours towards an ex-partner, such as constant texting, phone calling, letter-sending and following them (Douglas 2001)
- Domestic sexual violence, which indicates increased risk of serious physical injury, and a greater risk of sexual violence towards non-personally connected victims (‘stranger rape’) (Spencer 2020)
- A history of strangulation of a partner through choking, suffocation or drowning (Hester 2020)
- Making a credible threat to kill a partner (Spencer 2020)
- A history of weapon use (Folkes 2013)
- Coercive and controlling behaviour (Myhill 2019)

#### Attempts to accompany or speak for their partner/family member

People who are perpetrating domestic abuse will use opportunities to prevent the victim/survivor from speaking to others, to reduce opportunities for them to disclose the abuse. This is one reason why perpetrators may accompany victims/survivors who are patients of mental health services to appointments with healthcare professionals and/or be resistant to leaving a consultation to allow one-to-one assessment of the patient.

#### Excessive telephoning or texting/checking on their partner's whereabouts

Monitoring a person's whereabouts is a key tactic used by people perpetrating domestic abuse and it enables continued control of a victim/survivor. Indications of surveillance behaviour is an essential prompt for further enquiry.

#### Sexual jealousy or possessiveness, including delusional jealousy

Accusing a partner of cheating, or becoming angry when a partner speaks to people deemed a sexual/romantic threat, serve to increase the perpetrator's control of the victim/survivor. This behaviour may be accompanied by a clinical picture of delusional jealousy (Kingham 2004).

#### Substance use/dependence

Substance misuse is common among people who are using abusive behaviour in their family and intimate relationships. The combination of substance use with one or more of the other indicators in this list should suggest that further enquiry about domestic abuse is needed.

#### Suicidal behaviour/self-harm precipitated by their personal relationships

People who are perpetrating domestic abuse can threaten or attempt suicide as a strategy to maintain control in the relationship, particularly when it is jeopardised by separation (Kafka 2022). Professionals carrying out evaluations of risk in people presenting with suicidal ideation/behaviour should consider whether there are relationship stressors, including domestic abuse perpetration.

#### Injuries, especially hand injuries

Perpetrators of physical and sexual violence against partners or family members will sometimes incur physical injuries, especially where the victim/survivor attempts to resist. It is important to consider the possibility of abusive behaviour as an underlying factor if a person presents with minor physical injuries (such as hand injuries) and wider relationship difficulties.

#### Reporting of relationship issues

Although people using abusive behaviour in their intimate/family relationships may feel strongly that they are not to blame for the harm being caused, and/or may act to manipulate or blame the victim/survivor, expressing more general concerns or difficulties about their relationships is common and can generally be explored further with patients.

#### Threatening behaviour towards others, including staff

People who are perpetrating domestic abuse may also present a risk of harm to those to whom they are not personally connected, such as members of the public and professionals working in health and other services. Therefore, identification of violent behaviour towards health professionals should prompt detailed enquiries into other victims/survivors, including partners, children and other family members, as part of the developing clinical formulation and risk assessment.

### How to ask

There are many ways to explore what is happening in a patient's relationships in a neutral, curious and sensitive manner, in order to establish whether the patient is or may be perpetrating domestic abuse. Professionals should be alert to signs within the normal flow of a clinical encounter that should trigger further exploration, including signs of using coercive or controlling behaviour. A curious professional might explore current relationships in more detail if a patient reports that they are ‘struggling with managing anger’, for example when asked what happens when they argue with a partner/ex-partner/family member. Professionals may support someone to disclose by showing an understanding of the recognised consequences of a behaviour, for example by saying that sometimes when people are experiencing angry feelings, they may take this anger out on others around them.

Inquiry about perpetration of domestic abuse should be undertaken with victim/survivor safety as the core focus. This includes asking about harmful behaviour when the clinician is on their own with the patient, and after consideration of whether the environment is sufficiently private and safe for both the clinician and the patient to allow disclosure. Reminders should be given about duties to keep clinical information confidential, alongside duties to protect the patient, their family and the public. It can be helpful to present a rationale for asking these questions, which some patients may not consider to be directly relevant to their mental health.

Some individuals perpetrating domestic abuse will not disclose this despite direct and careful enquiry. It is possible that, through more frequent contact and developing a therapeutic relationship and rapport, disclosure of abusive behaviour might be made after more than one enquiry. Therefore, it is important to consider asking questions about perpetration of domestic abuse at regular intervals, where concerns/relevant indicators (as outlined elsewhere in this article) are present. It is also important to collect corroborative history from family members and partners.

### What to ask

Asking questions about a patient's use of harmful behaviour should be (where possible) collaborative, non-judgemental and focused on change. Examples could be:

- How are things in your relationships with your partner/family members?
- How do you deal with not getting on with your partner/family members?
- What happens when you argue?
- Has anything changed to make your relationship(s) more difficult?
- Do you ever feel worried about how you sometimes behave?
- Do arguments ever become physical, such as through pushing or hitting your partner/family member? What is the worst that has ever happened?
- How is this drinking/stress at work/depression affecting how you are with your family?
- When you feel like that, what do you do?

Identifying non-physical types of harmful behaviour such as coercive or controlling behaviour is important and needs specific questioning. Examples might be:

- Are you finding that you need to check on your partner (for example where they are, who they are with, what they are doing)?
- Do you ever check their mobile phone/tablet/laptop?
- Do you ever follow them when they go out (for example to check that they are OK)?
- Do you ever stop them from leaving the house when they say they want to?
- Have you ever stopped them from seeing friends or family?
- Have you ever stopped them from going to work or doing what they want to do because of how it makes you feel?
- Have you ever threatened to hurt yourself to stop them from doing something you didn't want them to do?
- Have you ever threatened them to stop them doing something you do not want them to do?
- Have you ever taken control of your partner's money, or stopped them spending money on things that they wanted?

## Responding to the disclosure or identification of domestic abuse perpetration

### Immediate considerations

Acting to reduce risk of serious harm to the victim/survivor should be the most important immediate consideration when perpetration of domestic abuse is identified or disclosed by a patient. Sharing information about a disclosure of domestic abuse perpetration with, for example, other family members, can increase risks. Where there is a disclosure of domestic abuse perpetration towards a specific person, it is important also to consider risk to other personally connected individuals, such as family members.

### Assessing risks in patients who are perpetrating domestic abuse

It is important to consider the level of risk of harm to the victim/survivor posed by a patient who is perpetrating domestic abuse. If there is immediate danger, practitioners should seek advice from safeguarding leads to explore whether to contact emergency services, updating the victim/survivor if possible/safe. Professionals who have identified that a patient is perpetrating domestic abuse should carry out and document a risk assessment in line with local policies and procedures if they feel confident to do so – that is, covering all relevant domains of risk (including risk to self; see below) (Royal College of Psychiatrists 2016).

Specific structured professional judgement tools such as the Domestic Abuse, Stalking and Harassment and Honour Based Violence Risk Identification, Assessment and Management Model (DASH), the Spousal Assault Risk Assessment (SARA), the Ontario Domestic Assault Risk Assessment (ODARA) or the Brief Spousal Assault Form for the Evaluation of Risk (B-SAFER) may be helpful to ensure that all relevant factors are addressed in the risk assessment, but they should be completed in collaboration with forensic and safeguarding staff with specific training in using these tools (Fazel 2018). Professionals should be able to access training in the risk assessment for domestic abuse perpetration and other forms of violent behaviour, to inform collaborative risk assessment within the multidisciplinary team (MDT). Factors identified in these tools that indicate greater risk of harm, including homicide, are described in Box 1.

Children should be considered victims/survivors of domestic abuse, regardless of whether they are present during abusive incidents. This is the case even if the victim/survivor does not recognise it. There are complex relationships between domestic abuse and violence towards children, with some evidence that perpetrators of domestic abuse may also perpetrate harm towards children. The harmful impact on children of being exposed to/witnessing/being aware of the domestic abuse is also well recognised. Therefore, risks towards children (specifically) posed by the perpetrator of domestic abuse should be assessed, including by asking the victim/survivor when they are on their own (and not in front of the perpetrator) if they are concerned about risks to children, and obtaining a full list of children in contact with the perpetrator. Family members or partners may be reluctant to disclose harm to children – there may be fear of social services action or of increased risk of abuse from the perpetrator if disclosures are made.

Refractory symptoms of mental illness such as delusions and hopelessness may be relevant to evaluating risk of future violence in perpetrators of domestic abuse. Ongoing misuse of alcohol and street drugs is related to greater risks. In addition to risk of harm to themselves, suicidal ideation and attempts at suicide by a perpetrator of domestic abuse indicate increased risk of perpetrating serious physical violence/homicide (Flynn 2016).

### Sharing information within the safeguarding team/health provider/trust

It is important to recognise that many cases of domestic abuse perpetration present substantial uncertainties and risks while not meeting criteria for onward referral and may present a clinical and risk management need that is not currently being met. Professionals should make contemporaneous records of clinical encounters and assessments in line with good medical practice, recording all information that is relevant to clinical care. Therefore, professionals should record suspicions of domestic abuse, even where they feel that they cannot be definitive – in these situations it is important to differentiate clearly between evidenced statements and impressions. Service managers can advise on where it is safe to document clinical information that the suspected perpetrator is unable to access. Safeguarding teams can advise on arrangements to share information on vulnerable individuals, including victims/survivors of domestic abuse. Professionals who identify that a crime has been committed, such as a physical assault as part of domestic abuse, or who witness a clear threat made against a named individual, should report their concerns to the police (e.g. in line with General Medical Council guidance) and make appropriate clinical documentation of this (e.g. recording a crime number in the notes). Reporting people perpetrating domestic abuse can introduce risks if it is done without the knowledge of the victim/survivor. Where possible, the professional, or a designated colleague professional such as an independent domestic violence advisor (IDVA), should speak to the victim/survivor prior to any reporting.

Mental health services are well placed to play a role in the coordinated community response (CCR) to domestic abuse (Shorey 2014). The CCR model acknowledges domestic abuse as a complex problem that has an impact on people, communities and systems across society, including health services, housing agencies and the criminal justice system (Robinson 2007). In this model, each agency that has a responsibility for dealing with perpetrators, their children and/or victims/survivors must work effectively internally and with all other agencies. This includes agencies taking appropriate actions within the remit of their role with victims/survivors and perpetrators, sharing information and working effectively and systematically with other services to increase the safety of victims/survivors and holding abusers to account.

The England and Wales Domestic Abuse Act 2021 includes provisions for domestic abuse protection orders (DAPOs), which can stipulate ‘positive requirements’ for domestic abuse perpetrators to engage with agencies such as substance misuse, mental health and behaviour change services at sentencing (Hester 2020). In addition, under the statutory Serious Violence Duty, all integrated care boards (ICBs) must collaborate across the care system to act to prevent and reduce serious violence, including domestic abuse (Hopkins 2022).

Where there are concerns that a perpetrator poses a high risk of harm to a victim/survivor, local safeguarding teams can advise regarding referring the case to a local multi-agency risk assessment conference (MARAC). A MARAC is a meeting at which information on the highest-risk domestic abuse cases is shared between local sector services, including the police, probation, health, child protection, housing practitioners and other specialists from the statutory and voluntary sectors. There will also be a representative from a local domestic abuse service, often an IDVA, who can connect victims/survivors to local specialist services if they want this support. Determinations of whether a case is high risk can be made based on professional judgement supported by multidisciplinary discussion, the completion of the DASH tool in collaboration with safeguarding staff (DASH advises MARAC referral where 14 or more boxes are ticked) or evidence of escalation in the number of police call-outs in the past year. However, there are no definitive thresholds for assigning ‘high risk’ categorisations to cases of domestic abuse. There may be considerable uncertainty regarding risk level, and all available information and content of MDT and interdisciplinary discussions should be documented.

Patients perpetrating domestic abuse may also meet criteria for referral to multi-agency public protection arrangements (MAPPA). These are statutory arrangements to assess and manage the risk posed by certain sexual and violent offenders, including ‘high-risk, high-harm’ and serial domestic abuse perpetrators. MAPPA were established by sections 325 to 327 of the Criminal Justice Act 2003. Local MAPPA guidance, the designated MAPPA lead and community forensic mental health services can advise further on suitability (Royal College of Psychiatrists 2013). Consultation with forensic psychiatry should be considered on a case-by-case basis, recognising that not all perpetrators of domestic abuse will be eligible for forensic mental health input or meet criteria for MAPPA discussion. Where the patient is discussed (as a victim/survivor and/or a perpetrator of domestic abuse) at a MARAC or under MAPPA it is usually local policy to highlight risk concerns in the form of a ‘risk alert’ in the patient record. In liaison with clinical managers, it may be wise to add alerts in patient records if you have significant risk concerns, even in the absence of referrals to multi-agency meetings. Previous evidence suggests that information sharing on perpetrators of domestic abuse within health services and with other agencies could be improved (Dheensa 2020).

### Address mental health conditions and/or comorbid substance use

Evidence on specific interventions to reduce violence by perpetrators of domestic abuse specific to people with mental health conditions is limited. One exception is in the context of substance use disorders, where behaviour change interventions may have benefit (Lila 2023). Evidence suggests that violent behaviour is pharmacologically modifiable in the context of established mental health conditions, including psychotic disorders (Bhavsar 2020; Fazel 2014). No evidence currently can inform on the effectiveness of psychological interventions for domestic abuse perpetration, although many perpetrator programmes incorporate cognitive–behavioural methods into group sessions.

## How identification and assessment of perpetrators relate to clinical management

Our framework (Fig. 1) reflects that perpetrators of domestic abuse identified in mental health services will usually be receiving therapeutic interventions for mental health and well-being, such as pharmacological prescribing, psychological therapies and social/occupational interventions. It may be necessary in this context to review and remove interventions once perpetration of domestic abuse is identified: for example, one-to-one anger management programmes or couples therapy are not considered appropriate interventions when domestic abuse is present.

### Interventions for perpetrators of domestic abuse

Perpetrator interventions are deliberately and intentionally designed to disrupt a perpetrator's use of abuse towards a victim/survivor and/or offer help to change. Specialist domestic abuse services for both victims/survivors and perpetrators are a key component of a CCR model.

There is geographical variation within the UK in resources available to perpetrators of domestic abuse who wish to change their behaviour. The range of interventions includes initial help-seeking services such as the national Respect Phoneline (respectphoneline.org.uk) and longer-term behaviour change work and intensive case management, ideally delivered by services that meet Respect or Home Office standards for perpetrator interventions (Respect 2022; Home Office 2023). The Respect Phoneline offers people concerned about their behaviour access to accredited support and direction to local behaviour change programmes. It provides honest, non-judgemental support and advice on addressing abusive behaviour. It also signposts callers to local behaviour change programmes. The Respect Phoneline can also provide health professionals with confidential advice and information on how to work safely with someone perpetrating or suspected of perpetrating domestic abuse.

Male perpetrators of domestic abuse may be referred to individual (one-to-one) or group-based perpetrator programmes (Hester 2019). These programmes are informed by behaviour change principles, a feminist perspective (emphasising the gender imbalance inherent in domestic abuse) and a focus on the definition of domestic abuse. Maintaining the safety of the victim/survivor is central to programme aims, and therapeutic/management work within these programmes aims to address and increase the accountability that the perpetrator takes for their abusive behaviour. Conducting randomised controlled trials to robustly evaluate the effectiveness of perpetrator programmes presents important challenges (Turner 2023). There is limited high-quality evidence for the effectiveness of perpetrator programmes, including for populations with mental health conditions, for female perpetrators and for perpetrators abusing non-partner family members. The priority outcome of any perpetrator interventions should be enhanced safety and freedom for all victims/survivors, including children.

Mental health professionals should consider their own personal safety in advance of any assessment for interventions (Royal College of Psychiatrists 2016). Patient records should be checked beforehand for relevant alerts, and arrangements made to assess with a colleague or to invite the patient to clinic rather than visiting them at home. Professionals should not attempt motivational or behaviour change work with perpetrators in the absence of specific training. It is important to consider straightforward health promotion advice for perpetrators of domestic abuse with mental health conditions, including advice on accessing social support and on sleep hygiene, diet, exercise and working habits. We also recognise that professionals may themselves have experienced domestic abuse, and they should ensure that they are accessing appropriate support where needed.

## Conclusions

Professionals working in mental health services have a duty to address domestic abuse. Clinicians possess the relevant skills and competencies to identify and respond to people perpetrating domestic abuse, as well as to victims/survivors. Any clinical response to the perpetration of domestic abuse must be rooted in a clear understanding of the dynamics of domestic abuse and best practice in this area, so that victim/survivor safety can be kept central to any response. We have suggested areas where specific knowledge and an approach to practice could improve the responses of individual clinicians, as well as those of mental health services more generally. Effective data collection is needed in clinical services to better understand the needs of this population, and rigorous research is required to further develop evidence-based guidance and interventions in this area. A range of resources both within mental health services and other agencies can offer expert advice on responses for this group.

## Data Availability

Data availability is not applicable to this article as no new data were created or analysed in this study.

## MCQs

Select the single best option for each question stem

1. What is the proportion of domestic homicide reviews in England where the perpetrator had received a mental health diagnosis?
  - 11%
  - 22%
  - 37%
  - 49%
  - 75%.
2. Which of the following is a risk assessment tool specifically for domestic abuse?
  - DASH
  - HCR-20
  - PCL-R
  - SAVRY
  - VRAG.
3. Which of the following interventions is *not* generally recommended in people with mental health conditions who are perpetrating domestic abuse?
  - Group-based behaviour change work
  - Individual behaviour change
  - Couples therapy
  - Antipsychotic prescribing
  - Calling 999.
4. Which of the following is *not* an indicator of risk included in risk assessments for domestic abuse perpetration?
  - The victim is afraid
  - A court case related to domestic abuse or child contact
  - Pregnancy of the partner
  - Non-adherence regarding prescribed oral medication
  - Strangulation of a partner.
5. Which of the following is *not* generally relevant to clinical responses in mental health services to the perpetration of domestic abuse:
  - The coordinated community response model
  - A multi-agency risk assessment conference
  - Multi-agency Public Protection Arrangements
  - The eco-social model
  - A child risk assessment.

MCQ answers 1 d 2 a 3 c 4 d 5 d
